# Are We Prepared in Case of a Possible Smallpox-Like Disease Emergence?

**DOI:** 10.3390/v9090242

**Published:** 2017-08-27

**Authors:** Victoria A. Olson, Sergei N. Shchelkunov

**Affiliations:** 1 Poxvirus and Rabies Branch, Division of High Consequence Pathogens and Pathology, National Center for Emerging and Zoonotic Infectious Diseases, Centers for Disease Control and Prevention, Atlanta, GA 30333, USA; 2 Department of Genomic Research and Development of DNA Diagnostics of Poxviruses, State Research Center of Virology and Biotechnology VECTOR, Koltsovo, 630559 Novosibirsk Region, Russia; 3 Department of Molecular Biology, Novosibirsk State University, 630090 Novosibirsk, Russia

**Keywords:** smallpox, *Variola virus*, antivirals, vaccine

## Abstract

Smallpox was the first human disease to be eradicated, through a concerted vaccination campaign led by the World Health Organization. Since its eradication, routine vaccination against smallpox has ceased, leaving the world population susceptible to disease caused by orthopoxviruses. In recent decades, reports of human disease from zoonotic orthopoxviruses have increased. Furthermore, multiple reports of newly identified poxviruses capable of causing human disease have occurred. These facts raise concerns regarding both the opportunity for these zoonotic orthopoxviruses to evolve and become a more severe public health issue, as well as the risk of *Variola virus* (the causative agent of smallpox) to be utilized as a bioterrorist weapon. The eradication of smallpox occurred prior to the development of the majority of modern virological and molecular biological techniques. Therefore, there is a considerable amount that is not understood regarding how this solely human pathogen interacts with its host. This paper briefly recounts the history and current status of diagnostic tools, vaccines, and anti-viral therapeutics for treatment of smallpox disease. The authors discuss the importance of further research to prepare the global community should a smallpox-like virus emerge.

## 1. Introduction

Implementation of the World Health Organization (WHO) Global Smallpox Eradication Program culminated in the last natural smallpox case, which was recorded 40 years ago in October of 1977. The subsequent efforts of the Global Commission for the Certification of Smallpox Eradication allowed the complete eradication of this disease to be stated [1]. The success of this program was first and foremost determined by the fact that *Variola virus* (VARV) is a solely human pathogen combined with the existence of an efficacious vaccine; live *Vaccinia virus* (VACV) vaccination provided reliable protection against this infection [1,2].

On 8 May 1980, the World Health Assembly declared smallpox eradicated. This is the first and still singular victory of the world community over a dangerous human infectious disease. Taking into account that the vaccination against smallpox in a proportion of the population had severe side effects, the WHO recommended a stop to widespread smallpox vaccinations in all countries after eradication [1]. As the result of this decision, humankind has lost immunity not only to smallpox, but also to other zoonotic, orthopoxvirus-caused human infections. In addition, remarkable advances in synthetic biology make it possible to de novo construct an *Orthopoxvirus*, as demonstrated recently with *Horsepox virus* [3], which increases the likelihood that VARV can be reconstructed for use in bioterrorist attacks. This fact, in combination with the increase in the rate of human infection with zoonotic orthopoxviruses observed recently [4,5,6,7,8,9,10,11,12,13,14,15,16,17,18,19,20,21,22,23,24], reinforces the importance of developing state-of-the-art methods for rapid and reliable, species-level identification of orthopoxviruses, designing safer smallpox vaccines, and characterizing antiviral chemotherapeutics addressed to different molecular targets.

## 2. Can a Smallpox-Like Disease Emerge?

VARV, a strictly human pathogen with a mortality rate in humans up to 40%, belongs to the genus *Orthopoxvirus* within the family *Poxviridae*. This genus also includes the following zoonotic viruses capable of causing infection within humans: *Monkeypox virus* (MPXV) [6,7,8,9], *Cowpox virus* (CPXV) [10,11,12], *Vaccinia virus* (VACV) [19,20,21], *Buffalopox virus* (BPXV, a subspecies of VACV) [16,17], and *Camelpox virus* (CMLV) [23].

MPXV causes the human infection that, in its clinical manifestations, resembles smallpox and has a case fatality rate of up to 10% [6,7,25]. The major difference between monkeypox in humans and smallpox is a low efficiency of person-to-person transmission, which so far has prevented expansion of local outbreaks to epidemics. However, recent studies suggest an increasing efficiency of MPXV spreading in the human population [8], causing concern for medical services in Central Africa, where the disease is enzootic.

Other zoonotic *Orthopoxvirus* species typically cause rare human infections (small outbreaks), mainly ending in a benign manner in healthy adults. However, CPXV infection in humans with immunodeficiency conditions can cause development of a generalized disease similar to smallpox with a lethal outcome [5,26,27]. Similarly, VACV infection can cause severe complications in persons with certain dermatologic conditions [1,2]. Although not traditionally considered a human pathogen, viable CMLV has recently been isolated from at least one infected camel herder [28].

The eradication of smallpox brought into question whether natural evolution could lead to the transformation of existing zoonotic orthopoxviruses to VARV. The first step in the attempt to answer this question consisted of genome sequencing and comprehensive analysis of various VARV [29,30,31,32,33,34,35], MPXV [36,37,38], CPXV [39,40,41], VACV [42,43], and CMLV [44,45] strains. As has emerged, the CPXV genome is the largest and comprises all genes characteristic of the other *Orthopoxvirus* species [39]. Orthologs of several genes in the genomes of the remaining species of orthopoxviruses are either truncated or deleted, so that they differ in the set of the retained genes [2,37,39,46,47,48,49]. These data favor the concept of a reductive evolution of orthopoxviruses, implying that gene loss plays an important role in the evolutionary adaptation of an ancestor virus to a particular environmental niche (host) and the emergence of new virus species [50,51]. VARV, the virus most pathogenic for humans, has the smallest genome of all orthopoxviruses [2,37]. Thus, it is theoretically possible for a VARV-like virus to emerge via natural evolution from the currently existing zoonotic orthopoxviruses that have a larger genome [4,52].

Orthopoxviruses contain double stranded DNA genomes which do not mutate as quickly as RNA viruses (e.g., influenza) but are known to evolve over time. Phylogenetic analysis of the genomes of a large set of VARV strains isolated between 1944–1977 shows that the VARV isolates from South America and West Africa are distinct from each other but cluster in a common clade considerably different from the remaining examined VARV isolates that originated from other geographical regions [53]. This suggests that the West African VARV variants were imported to South America and allowed dating to be introduced to phylogenetic analysis and the rate of poxvirus molecular evolution to be assessed [54,55,56,57]. Recent genomic sequencing of one VARV isolate from a mummy of the 17th century suggests an abbreviated timescale of VARV evolution [58].

One hypothesis proposes smallpox could have repeatedly emerged by way of evolutionary changes of an ancestral zoonotic virus and disappeared because of insufficient population density due to the separated ancient civilizations. This hypothesis was formulated based on the available archival records concerning smallpox epidemics, historical data on ancient civilizations, and newer data on evolutionary relationships of orthopoxviruses [52]. There are no fundamental prohibitions for the emergence of smallpox or a similar human disease through natural evolution of currently existing zoonotic orthopoxviruses. Thus, it is necessary to develop and widely adopt state-of-the-art methods for efficient and rapid, species-specific diagnosis of all orthopoxviruses that are pathogenic for humans, VARV included. It is also necessary to develop new, safe methods for the prevention and therapy of human *Orthopoxvirus* infections. Two WHO Collaborating Centers that are authorized as repositories of VARV—Centers for Disease Control and Prevention (CDC), Atlanta, Georgia, United States of America, and State Research Center of Virology and Biotechnology (VECTOR), Koltsovo, Novosibirsk region, Russian Federation—play key roles in research for preparation should a smallpox-like disease emerge.

## 3. Methods for Species-Specific Diagnosis of Orthopoxviruses

A multicomponent strategy of surveillance and containment is recommended in the case of a smallpox outbreak [1]. A critical component of this strategy is the early detection of a smallpox case. A delay in detection is likely to have a considerable impact on the overall control strategy. *Orthopoxvirus* infections cause clinically distinctive skin lesions, yet they can be misdiagnosed. Resources available on WHO [59] and CDC [60] websites provide guidance to clinicians in recognizing smallpox disease, yet the clinical descriptions and photographs have limitations for training. The likelihood of misdiagnosis only increases as time elapses since the last case of smallpox. Very few practicing clinicians today have ever seen patients with smallpox lesions. To improve early detection, the main requirements for laboratory diagnostic tests are rapid results with high sensitivity and specificity.

The advent of DNA fragment amplification by polymerase chain reaction (PCR) [61,62] formed the background for designing various techniques appropriate for rapid identification of orthopoxviruses. PCR-based DNA fragment amplification makes it possible to recover the specific DNA fragments from trace quantities of genetic material in clinical samples without the need to cultivate the virus. This rapid direct diagnostic method is especially important to limit manipulations of samples containing highly pathogenic strains.

Determination of the genome nucleotide sequences for a number of strains of several *Orthopoxvirus* species [29,30,31,32,33,34,35,36,37,38,39,40,41,42,43,44,45] revealed species-specific differences at certain loci, allowing development of new methods for virus identification. The very first PCR-based diagnostic assays for the detection of orthopoxviruses pathogenic for humans utilized oligonucleotide primers directed against conserved regions of the genes encoding hemagglutinin [63], type-A inclusion body protein [64], or the homolog of tumor necrosis factor receptor [65]. In all these studies, the DNA fragments produced by PCR were hydrolyzed by specific restriction endonucleases and separated by electrophoresis (PCR-Restriction Fragment Length Polymorphism (PCR-RFLP)), which allowed for species-specific identification of orthopoxviruses according to the pattern of subfragments. However, analysis of a sufficiently large set of isolates of an *Orthopoxvirus* species (especially CPXV) demonstrated their heterogeneity in the set of produced subfragments, making the data interpretation rather ambiguous.

Multiplex PCR analysis of the human-pathogenic orthopoxviruses, an alternative to the PCR-RFLP approach, makes it possible to increase the degree of specificity. In this method, unique oligonucleotide primers for the genetic loci individual for each orthopoxvirus species allow for species-specific identification in one stage. All five pairs of primers (for VARV, MPXV, CPXV, and VACV, as well as one genus-generic pair) are used in one PCR reaction generating the products of different sizes characteristic of each species [66].

Real-time PCR (RT-PCR) assays allow identification of the amplified products with the help of fluorescent dyes. Exclusion of electrophoresis makes the assay more rapid, and considerably decreases the potential for contamination of samples. Various RT-PCR combinations were used in the design of the methods for the detection and species-specific identification of orthopoxviruses. A large set of methods providing the detection of orthopoxviruses have been elaborated so far; mainly, they utilize TaqMan and LightCycler (Roche Indianapolis, IN, USA) variants of RT-PCR [67,68,69,70,71,72,73,74,75,76,77,78,79,80,81,82,83,84,85]. Several of these assays have undergone review by regulatory agencies for diagnostic use. Within the United States, two RT-PCR assays which detect VARV and one RT-PCR assay, which detects *Orthopoxvirus,* excluding VARV, have been approved by the Food and Drug Administration. Note that these procedures are able to identify viruses to a genus level and differentiate only one of the *Orthopoxvirus* species: VARV, MPXV, CPXV, or VACV. However, a method allowing simultaneous differentiation of VARV, MPXV, CPXV, and VACV in the same RT-PCR reaction mixture has been recently developed. A kit using this more universal approach, with a high potential for wide application [86], was officially approved in Russia for clinical use in 2016.

As technology advances, diagnostic techniques must adapt and improve. One technologic enhancement has been the development of a portable real-time PCR platform, allowing diagnostic assays to be conducted in endemic areas. The GeneXpert platform (Cepheid Sunnyvale, CA, USA) is currently being used for diagnosis of monkeypox within surveillance specimens from the Democratic Republic of Congo. The multiplex assay includes a MPXV-specific assay, orthopoxvirus-generic assay, and an internal control within the same cartridge [87]. The methodology decreases manipulations of the samples, thereby also limiting opportunities for contamination. A deployable, point-of-care diagnostic assay will minimize the time for the confirmation of human infections of *Orthopoxvirus* disease, a crucial component should an outbreak occur.

Another advancement is the oligonucleotide microarray, which utilizes PCR with subsequent species identification by hybridization of fluorescently labeled, amplified products to specific oligonucleotides immobilized in a certain order on a carrier. By analogy to classic PCR, this method is able to detect trace quantities of the analyzed DNA in a sample and has been utilized to design a set of microarrays for species-specific identification of orthopoxviruses [88,89,90,91]. One of the advantages of oligonucleotide microarrays over other PCR-based diagnostic methods is the possibility to simultaneously analyze many loci, which considerably increases confidence in diagnosis based on the microarray method. The technology of DNA microarrays is promising for clinical diagnosis aimed at identification and differentiation of infectious agents. However, the equipment and technology for microarrays are still only available in advanced research laboratories, being out of reach for ordinary diagnostic laboratories.

The emergence of new *Orthopoxvirus* isolates presents an ever-present complication for the reliable determination of which *Orthopoxvirus* species is causing an infection. In recent years, new orthopoxviruses pathogenic to humans have been reported [92,93]. Akhmeta virus was isolated in cattle herders within Georgia and a novel *Orthopoxvirus* was isolated from an Alaskan resident. Rapid development of sequencing technologies now allows for a full genomic sequence to be obtained from these isolates quickly. These new species may confound current diagnostic assays thought to be species-specific [40,83,92], reinforcing the need to be ever vigilant in validation of current diagnostic techniques. These studies demonstrate that it is necessary to continue the improvement of laboratory diagnosis for *Orthopoxvirus* infections and epidemiologic surveillance. The human-pathogenic zoonotic orthopoxviruses circulating in nature require comprehensive study; monitoring of the emergence of new species [92,93] is also necessary, since their appearance in the background of ceased vaccination against smallpox can theoretically lead to the emergence of *Orthopoxvirus* variants highly pathogenic for humans.

## 4. Modern Vaccines Against Smallpox

The vaccine used to eradicate smallpox was a “first-generation” vaccine consisting of a VACV preparation produced by reproducing the virus on the skin of calves or other animals [1], a crude method of preparation which would not satisfy regulatory authorities today. First generation vaccines were known to cause serious adverse events in certain individuals during the eradication era [1,2]. Therefore, considerable focus centered on developing safer vaccines against smallpox. Currently, VACV vaccine strains are reproduced in mammalian cell cultures; these preparations are regarded as second-generation vaccines against smallpox [94].

The first-generation vaccine preparations were heterogeneous [95,96,97], while the homogeneous second-generation vaccines contain clonal isolates of VACV. The clonal variants for the production of second-generation vaccines should be selected from heterogeneous preparations with several characteristics compared in parallel, such as the safety and immunogenicity of the vaccine clone. Since smallpox has been eradicated and an animal model that replicates human disease is not available (see Section 6), the assessment of the efficacy of these second-generation vaccines must rely upon other measures. One method to test the vaccine efficacy relative to the traditional first-generation vaccines against smallpox uses various smallpox surrogate animal models; in particular, mousepox in laboratory mice infected with *Ectromelia virus* (ECTV), rabbitpox in rabbits infected with *Rabbitpox virus* (RPXV, a VACV subspecies), or monkeypox in nonhuman primates infected with MPXV. An independent approach is a comparative assessment of the ability of virus-neutralizing antibodies to inactivate orthopoxviruses, including in vitro confirmation of VARV neutralization [98].

To generate the ACAM2000 (Sanofi Pasteur, Lyon, France) clonal vaccine, licensed in the United States in 2007, 30 ampoules of the smallpox vaccine Dryvax (Wyeth, Philadelphia, PA, USA) from three production batches were pooled and cloned in the MRC-5 cell line; six independent clones were obtained [99]. Further experiments demonstrated that only three clones of these six induced characteristic lesions on the rabbit skin comparable to Dryvax and displayed comparable or reduced neurovirulence after intracerebral administration of virus to adult mice. Candidate clone ACAM1000 demonstrated decreased virulence compared with Dryvax, yet ACAM1000 and Dryvax displayed comparable results on immunogenicity in animals and humans [96]. The MRC-5 cell line demonstrated a low efficiency in virus production so the Vero cell line on microcarriers in bioreactors with a serum-free medium was used for the vaccine production of ACAM1000; the final product was named ACAM2000 [100,101]. Further clinical trials demonstrated that, although ACAM2000 had decreased neurovirulence in animals, vaccination still induced a high rate of side effects in vaccinees (similar to Dryvax), including myocarditis at a rate of one case per 145 primary vaccinees [102,103]. Thus, although cell culture-based manufacture of vaccines meets modern standards, the second-generation vaccines can nonetheless cause serious post-vaccination side effects similar to the first-generation vaccines and, thus, their use is limited [103].

To achieve safer vaccines, third-generation-attenuated vaccines against smallpox are created by multiple passages of a certain VACV strain in a heterologous host cell culture. For example, Modified Vaccinia Ankara (MVA), the best-studied third-generation smallpox vaccine, was obtained by 572 passages of the VACV strain Ankara in chick embryo fibroblast culture. Numerous mutations and extended deletions relative to the initial strain emerged in the MVA genome. Characteristic of MVA is its inability to replicate in most mammalian cells, including human cells [104]. So far, the vaccine based on VACV strain MVA (Imvanex/Imvamune) produced by Bavarian Nordic (Kvistgaard, Denmark) has passed 19 clinical trials involving a total of 7676 volunteers, including 1070 atopic dermatitis and HIV positive subjects [105,106,107,108]. The vaccine induces the profile of antibodies analogous to that induced by the classic first-generation vaccine and protects against zoonotic orthopoxviruses. This has been shown in various laboratory animal models, such as MPXV-infected rhesus macaques, RPXV-infected rabbits, and VACV-infected mice [109,110,111]. Imvanex/Imvamune has been licensed for exposure to smallpox in 32 countries, including all European countries and Canada. Data, to include the in vitro VARV neutralization ability of vaccinee sera, are being generated for submission to the Food and Drug Administration for licensure within the United States. The first and foremost intended use of MVA would be for the primary vaccination of individuals with contraindications to the first- and second-generation smallpox vaccines.

Another third-generation vaccine against smallpox, LC16m8 (Kaketsuken, Kumamoto, Japan), is licensed in Japan. LC16m8 vaccine was produced by passaging the VACV strain Lister 45 times in primary rabbit kidney cell culture at a decreased temperature (30 °C). Clinical trials have demonstrated a considerable decrease in the rate of side effects as compared with the traditional vaccine involving VACV strain Lister [112]. A mutation (deletion) in the *B5R* gene, encoding a protein on the Extracellular Enveloped Virus (EEV—one of the two infectious forms of orthopoxviruses progeny), is the major factor explaining attenuation of LC16m8 [112,113]. The protective effect of LC16m8, as comparable to the parental VACV strain Lister, has been demonstrated in manifold experiments with both immunocompetent and immunodeficient mice [114], rabbits [115], and nonhuman primates [116].

A novel approach to producing the fourth-generation attenuated vaccine against smallpox consists of introducing directed deletions/insertions interfering with the viral immunomodulatory genes, such as those that control the host’s protection against viral infection, and the range of sensitive hosts. The VACV strain NYVAC is the best-studied example, having a block of 12 genes deleted in its genome and an additional six genes impaired [117]. As has been shown, NYVAC induces lower levels of neutralizing antibodies in humans as compared with the classic VACV Lister strain-based vaccine or Dryvax. NYVAC is unable to induce A27-specific neutralizing antibodies, which are necessary for efficient neutralization of Intracellular Mature Virus (IMV—one of the two infectious forms of orthopoxviruses progeny) [118].

Research continues to identify directed mutations that will provide a safe and efficacious vaccine. A highly attenuated VACV strain was obtained in Russia by successive introduction of directed deletions/insertions in five individual genes of the LIVP (Lister strain variant of Institute for Vaccine Preparations, Moscow, Russia) strain [119]. An additional directed deletion introduced into the *A35R* gene of the LIVP genome gave a highly immunogenic-attenuated strain, VACdelta6 [120], which is now undergoing preclinical trials as a fourth-generation vaccine candidate.

## 5. Anti-Smallpox Chemotherapeutics

The primary objective of preparedness is to save lives if smallpox somehow emerges. The limited historic reports that smallpox vaccination post-exposure was able to prevent smallpox have been challenged by more recent animal studies [121]. Thus, the development of antiviral therapies is important for outbreak response efforts as well as in disease treatment. Currently there are no drugs approved for use in the treatment of poxvirus infections, and this is an area of active research and development [122]. Ideally, multiple compounds targeting distinct mechanisms of the *Orthopoxvirus* life cycle would be incorporated into the treatment regimen to minimize morbidity and mortality.

A multitude of compounds have been evaluated for their ability to halt *Orthopoxvirus* replication. These include compounds which target host tyrosine kinases [123], viral DNA replication [124,125], viral transcription [126,127], and viral morphogenesis [128,129,130,131,132,133]. Amongst these compounds, two are at late stages of development.

The first is an inhibitor of poxvirus egress, the experimental drug tecovirimat (TPOXX^®^-formerly known as ST-246, SIGA Technologies, Inc., New York, NY, USA). Considerable in vitro and in vivo experimentation has demonstrated its effectiveness against orthopoxviruses, including VARV and MPXV infections of non-human primates [128,129,132,134]. A similar chemically synthesized compound NIOCH-14 (Novosibirsk Institute of Organic Chemistry, Novosibirsk, Russia) has shown equivalent protection of marmots challenged with MPXV and SCID mice challenged with VARV compared to TPOXX^®^ [133]. Clinical trials have shown TPOXX^®^ to have a strong safety profile [135] and, in fact, the compound has been used investigationally in the treatment of human smallpox (vaccinia) vaccine-adverse events in the United States [136,137].

The second compound in late stages of development is the DNA polymerase inhibitor. Brincidofovir (Chimerix, Durham, NC, USA), the orally bioavailable derivative of cidofovir, has increased potency with no evidence of the nephrotoxicity seen with cidofovir treatment [138]. Similar to TPOXX^®^, brincidofovir has considerable in vitro and in vivo data to support anti-orthopoxvirus activity, particularly against RPXV and ECTV in vivo [139,140]. Brincidofovir is unique in that it targets multiple DNA viruses and is in development for treatment of infections with cytomegalovirus, adenovirus, and orthopoxviruses [138,141]. Initial Phase I clinical trial data suggests brincidofovir would have a favorable safety profile for the treatment of smallpox [142]. Along with TPOXX^®^, cidofovir and/or brincidofovir were also used investigationally in the treatment of human smallpox (vaccinia) vaccine-adverse events in the United States [136,137].

Although these compounds have promise, there are still limitations to their effectiveness. Despite their efficacy, mutations within orthopoxviruses have arose that provide resistance to these compounds [137,143]. In fact, during elongated treatment of a patient with progressive vaccinia, TPOXX^®^ resistant isolates developed. Since only a few (1–5) amino acid changes may be sufficient to circumvent the blockade provided by these compounds [143,144], it is prudent to continue development of additional compounds as potential therapeutics for *Orthopoxvirus* disease. These data also reinforce the utility of an anti-viral therapeutic regimen which utilizes multiple compounds targeting various stages of the *Orthopoxvirus* life cycle.

A different type of anti-viral therapy relies upon the transfer of neutralizing antibodies. Although data is limited, historic reports suggest that treatment with immune products from persons vaccinated against smallpox or survivors of VARV infection may provide protection against smallpox [145,146,147]. Vaccinia immune globulin (VIG) is a licensed product in the United States for use as a treatment for severe adverse events associated with smallpox vaccination. However, VIG is in limited supply since it is no longer mass produced after the eradication of smallpox and the subsequent end of widespread smallpox vaccinations. In recent years, hybridoma technology has significantly advanced, allowing for the production of human antibodies isolated from peripheral blood mononuclear cells. These human antibodies derived from vaccinees or survivors of disease have been shown to be effective at halting the progression of multiple orthopoxviruses’ life cycle in vitro [148]. Moreover, these antibodies demonstrate greater protection in vivo against VACV compared to VIG. These renewable resources show great promise as another potential anti-viral therapy that may minimize morbidity and mortality.

## 6. Animal Models of Human Smallpox

As noted above, the development of medical countermeasures (MCMs) against smallpox has focused on safer vaccines and compounds with anti-viral activity. The use of novel compounds will require extensive testing for the safety profile, for efficacy against VARV, and for the characterization of the mechanism of action. Critical steps to evaluate such therapeutics require both in vitro and/or animal model characterization of their activity, preferably against live VARV. Since VARV is solely a human pathogen and smallpox is eradicated, a constant limitation for the evaluation of MCMs is the inability to measure their efficacy in vivo. Multiple animal species have been investigated for their potential to mimic human smallpox. It is highly unlikely that one animal will be capable of demonstrating disease progression similar to human smallpox upon VARV infection. More likely is the idea that different animal models of VARV infection will provide different methods to test the efficacy of the MCM.

An ideal animal model for the study of a human disease is one which utilizes a route, a dose, and presents a disease course similar to that seen within humans. Macaques have been used as an animal model for VARV infection with multiple successes. VARV infection of macaques causes disseminated classic smallpox lesions and can mimic hemorrhagic smallpox disease [25,149]. However, the macaque VARV model also has considerable limitations. Macaques require a high dose of infectious VARV delivered intravenously to cause lesional disease or hemorrhagic smallpox. This unnatural route and dose bypasses the typical incubation period noted for smallpox patients after natural routes of infection. Due to this abbreviated disease course, evaluations of anti-viral compounds must be extrapolated in regards to the onset and length of treatment. Furthermore, there are inherent challenges and limitations to experiments using macaques in regards to animal numbers, cost, and manipulations when working in the biosafety level 4 laboratory, which is required for studies using live VARV.

To overcome some of these challenges, smaller laboratory animals have been examined for susceptibility to VARV infection. Historically, adult mice have been noted to be insusceptible to VARV, similar to what is also seen with MPXV infection [150]. A screen of 38 mouse strains identified one that was highly susceptible to MPXV, the CAST/EiJ mouse [151]. However, when this mouse strain was exposed to VARV, very minimal viable virus was shed and the animals did not display any signs of morbidity. Investigations using another mouse strain (the ICR strain) have identified productive VARV replication within nasal and lung tissues [152]. These mice may demonstrate utility in determining the efficacy of potential MCMs by monitoring viral load within the infected mice, although clinical signs of disease are absent. The prairie dog (*Cynomys ludovicianus*) [153,154,155] and ground squirrel (*Marmota bobak*) [156] are small animal models that demonstrated disease progression which closely mimics human monkeypox and has been instrumental in the evaluation of therapeutics against MPXV. Unfortunately, despite the susceptibility to MPXV, the prairie dog displayed resistance to VARV infection [157].

More recently, animal model investigations of other solely human pathogens, such as HIV, Dengue virus, Epstein-Barr virus, and Ebola virus, have been successful utilizing the humanized mouse model [158,159,160,161]. Three different strains of humanized mouse were evaluated for susceptibility to VARV infection. Although none of the animals developed a disseminated rash or shed high viral loads from the oral cavity, mortality was seen in a dose dependent manner with high viral titers in all tissues sampled. The animals displayed an incubation period of approximately 13 days before the onset of symptoms, similar to human smallpox. These attributes make this animal model a prospect for the evaluation of potential anti-viral compounds and the determination of onset and length of treatment. Although one ideal animal model has not been identified that recapitulates human smallpox, different models of VARV infection have qualities which will allow them to be instrumental in the evaluation of potential MCMs (safer vaccines and anti-viral therapeutics).

## 7. WHO Collaborating Centers

The WHO played a key role in smallpox eradication. In order to minimize the threat of VARV release into the environment, the number of laboratories that retain the collections of VARV isolates was reduced to two WHO Collaborating Centers, namely, Centers for Disease Control and Prevention, Atlanta, Georgia, United States of America, and State Research Center of Virology and Biotechnology VECTOR, Koltsovo, Novosibirsk region, Russian Federation. These WHO Collaborating Centers ensure the conditions for the highest degree of physical protection, which is required for manipulations with live VARV. WHO international commissions on a regular basis (biennially) inspect both facilities to ensure the biosafety and biosecurity requirements are met. In addition, any study involving live VARV must first be proposed to the WHO Advisory Committee on *Variola Virus* Research (ACVVR). Studies can only be initiated after WHO’s official approval is granted. Since 1999, the WHO ACVVR has held annual meetings to review progress on VARV research projects and consider plans for further studies essential for public health, such as improvements for smallpox diagnostics, safe vaccines, and anti-viral therapies [162].

Since the inception of WHO review of studies has focused on live VARV, there has been considerable progress achieved. In December 1990, the WHO Ad Hoc Committee on *Orthopoxvirus* Infections approved studies for American and Russian scientists to complete genome sequencing of selected VARV strains. Multiple VARV genomes [29,30,31,32,33,34,35] have been sequenced along with other orthopoxviruses (ex. CPXV [39,40], MPXV [36,37,38], Ahkmetapox [82]) and comprehensively analyzed at the WHO Collaborating Centers [29,30,31,32,33,34,35,36,37,38,39,40,46,47,48,49,53,55]. These data allow for a deeper phylogenetic analysis and reveal the major evolutionary patterns of the orthopoxviruses pathogenic for humans [54,55,56,57]. The accumulated huge volume of data on the nucleotide sequences of various strains of VARV and other poxviruses form the background for designing manifold methods for reliable DNA diagnostics for these viruses (see Section 3).

Considerable effort has focused on creating safer vaccines for the prevention of smallpox (see Section 4). However, these vaccines were not in use while smallpox was endemic, and therefore their efficacy to prevent disease is unknown. In the absence of a reliable animal model to recapitulate human smallpox (see Section 6), the efforts of WHO Collaborating Centers to verify the in vitro VARV neutralization by sera from new generation smallpox vaccinees are of the utmost importance.

Currently, there is not an anti-viral therapeutic licensed for the treatment of orthopoxviruses. There has been a great deal of data generated on two candidate compounds that are in the late stage of development (see Section 5). However, these compounds still have limitations since small alterations to the *Orthopoxvirus* genome may provide resistance to these compounds. It is imperative to continue research on other compounds, as well as other techniques, to enhance our repertoire of anti-viral therapies. With a more robust inventory, the global community will be better prepared should a smallpox-like virus emerge.

In general, human *Orthopoxvirus* infections are monitored under the aegis of WHO and international experts on a regular basis. The WHO ACVVR reinforces that the most important areas for research with live VARV are universally available methods for smallpox diagnosis, prevention, and therapy. The WHO collaborating centers are committed to furthering research in support of these immediate public health goals. There are significant tools available to the world community should a smallpox-like disease emerge; however, continued research into orthopoxviruses, VARV included, is necessary. Additional understanding of *Orthopoxvirus* disease will ensure we retain capabilities to detect and mitigate disease, and will additionally improve our preparedness for the unknown.
